# Response to Hossain and Others: Hospital-Based Surveillance for Japanese Encephalitis at Four Sites in Bangladesh, 2003–2005

**DOI:** 10.4269/ajtmh.2010.10-0130a

**Published:** 2010-08-05

Dear Sir:

In response to Hossain and others^1^ paper on hospital based surveillance confirming the presence of Japanese Encephalitis (JE) in Bangladesh. We comment on the small proportion of confirmed cases of JE and the need to clarify sample timing of sample relative to onset of illness to confirm absence of JE virus in the enzyme-linked immunosorbent assay (ELISA) negative cases.

Hossain and others'^1^ paper on hospital-based surveillance for JE in Bangladesh provides important data on the spread of this devastating disease.^1^ This paper is important, because it shows further evidence of JE in Bangladesh, previously only reported during an outbreak in 1977.^2^

They systematically assessed an impressive 2,609 patients over four study sites, with 492 meeting their criteria for screening for JE virus (JEV). Of the cases of acute encephalitis (AES), their results confirm that 4% had JEV. It is also important to consider the patients with negative ELISA results for JEV; 472 (96%) of the patients had an unknown cause of their AES. ELISA testing is the method of choice for JEV, because viraemia is too transient for molecular methods, such as polymerase chain reaction, to be useful. There are data that show that IgM responses to JEV take between 7 and 10 days to develop.^3^,^4^ However, there are also data to suggest that an early low cerebrospinal fluid (CSF) immunoglobulin (Ig)M response may be suggestive of a poor outcome^5^; thus, if the CSF IgM rise is only transient, it may be missed by single sampling. Accurate diagnostics in the field are notoriously difficult, but it is crucial that this problem is highlighted and that timing of samples is clearly described so as not to engender complacency about the real prevalence and incidence of JE in cases of AES.

The current World Health Organization (WHO) JE surveillance standards recommend a sample at ≥ 10 day of illness to confirm or exclude JEV.^6^ Patients with early JEV ELISA negative samples should, thus, not be classified as JE negative but instead, as having JEV unknown status. Although it can be difficult to get the timing of the illness onset, it is preferable, where possible, to distinguish between JEV negative and JEV unknown, so that the JEV unknown cases can be excluded from any calculations of the proportion of cases caused by JEV. These data are helpful in informing government and funding bodies as to where to target JE vaccination programs.

Penny Lewthwaite

Brain Infections Group

University of Liverpool, Liverpool

United Kingdom

E-mail: pennylewthwaite@doctors.org.uk

Tom Solomon

Brain Infectious Group and Division of Neurological Science

University of Liverpool

Walton Centre for Neurology and Neurosurgery

Liverpool, United Kingdom

## References

[1] Hossain MJ Gurley ES Montgomery S Petersen L Sejvar J Fischer M Panella A Powers AM Nahar N Rafique Uddin AK Rahman ME Saifuddin Ekram AR Luby SP Breiman RF 2010 Hospital-based surveillance for Japanese encephalitis at four sites in Bangladesh, 2003–2005 Am J Trop Med Hyg 82 344 349 20134015 10.4269/ajtmh.2010.09-0125 PMC2813179

[2] Khan AM Khan AQ Dobrzynski L Joshi GP Myat A 1981 A Japanese encephalitis focus in Bangladesh J Trop Med Hyg 84 41 44 6259374

[3] Burke DS Nisalak A Ussery MA Laorakpongse T Chantavibul S 1985 Kinetics of IgM and IgG responses to Japanese encephalitis virus in human serum and cerebrospinal fluid J Infect Dis 151 1093 1099 2987367 10.1093/infdis/151.6.1093

[4] Solomon T Thao LT Dung NM Kneen R Hung NT Nisalak A Vaughn DW Farrar J Hien TT White NJ Cardosa MJ 1998 Rapid diagnosis of Japanese encephalitis by using an immunoglobulin M dot enzyme immunoassay J Clin Microbiol 36 2030 2034 9650956 10.1128/jcm.36.7.2030-2034.1998 PMC104972

[5] Libraty DH Nisalak A Endy TP Suntayakorn S Vaughn DW Innis BL 2002 Clinical and immunological risk factors for severe disease in Japanese encephalitis Trans R Soc Trop Med Hyg 96 173 178 12055808 10.1016/s0035-9203(02)90294-4

[6] World Health Organization 2006 WHO Recommended Standards for Surveillance of Selected Vaccine-Preventable Diseases Geneva, Switzerland World Health Organization

